# The 3D Printability and Mechanical Properties of Polyhydroxybutyrate (PHB) as Additives in Urethane Dimethacrylate (UDMA) Blends Polymer for Medical Application

**DOI:** 10.3390/polym14214518

**Published:** 2022-10-25

**Authors:** Ahmad Adnan Abu Bakar, Muhammad Zulhilmi Zainuddin, Shahino Mah Abdullah, Nizam Tamchek, Ikhwan Syafiq Mohd Noor, Muhammad Syafiq Alauddin, Ahmad Alforidi, Mohd Ifwat Mohd Ghazali

**Affiliations:** 1 SMART RG, Faculty of Science and Technology (FST), Universiti Sains Islam Malaysia (USIM), Nilai 71800, Malaysia; 2 Department of Physics, Faculty of Science, Universiti Putra Malaysia (UPM), Serdang 43400, Malaysia; 3 Physics Division, Centre of Foundation Studies for Agricultural Science, Universiti Putra Malaysia, Serdang 43400, Malaysia; 4 Department of Conservative Dentistry and Prosthodontics, Faculty of Dentistry, Universiti Sains Islam Malaysia, Kuala Lumpur 55100, Malaysia; 5 Electrical Engineering Department, Taibah University, Medina 42353, Saudi Arabia

**Keywords:** additive manufacturing, 3D printing, polyhydroxybutyrate, mechanical properties, medical application

## Abstract

The integration of additive manufacturing (3D printing) in the biomedical sector required material to portray a holistic characteristic in terms of printability, biocompatibility, degradability, and mechanical properties. This research aims to evaluate the 3D printability and mechanical properties of polyhydroxybutyrate (PHB) as additives in the urethane dimethacrylate (UDMA) based resin and its potential for medical applications. The printability of the PHB/UDMA resin blends was limited to 11 wt.% as it reached the maximum viscosity value at 2188 cP. Two-way analysis of variance (ANOVA) was also conducted to assess the significant effect of the varied PHB (wt.%) incorporation within UDMA resin, and the aging duration of 3D printed PHB/UDMA on mechanical properties in terms of tensile and impact properties. Meanwhile, the increasing crystallinity index (CI) of X-ray diffraction (XRD) in the 3D printed PHB/UDMA as the PHB loading increased, indicating that there is a strong correlation with the lower tensile and impact strength. FESEM images also proved that the agglomerations that occurred within the UDMA matrix had affected the mechanical performance of 3D printed PHB/UDMA. Nonetheless, the thermal stability of the 3D printed PHB/UDMA had only a slight deviation from the 3D printed UDMA since it had better thermal processability.

## 1. Introduction

In recent decades, a wide range of biopolymers has stimulated researchers’ interest to explore and utilize their benefits to create a sustainable material for the sake of environmental issues. Polyhydroxyalkanoates (PHA) are those among biopolymers that can act as an alternative to the synthetic polymers derived from petrochemicals [1]. PHA are biopolymers that are naturally derived and accumulated by different families of microorganisms such as Azotobacter, Bacillus and Pseudomonas [2,3,4]. PHA are classified according to their basic structural chain length: short chain length (consists of 3–5 carbons), medium chain length (6–14 carbons) and lastly long chain length (consists of more than 15 carbons) [5]. Polyhydroxybutyrate (PHB) falls under short chain length in the PHA family and is also the most common form of PHA [6].

Polyhydroxybutyrate (PHB) is a microbial aliphatic polyester created through the deprivation of nitrogen, phosphorus, or oxygen in the availability of excess carbon sources [7]. Highly crystallized PHB due to its stereo-chemical regularity of the structure contributed towards its excellent mechanical properties; high elasticity of modulus ranging between 3 to 3.5 GPa and tensile strength of 20–40 MPa [8]. In addition, its water-insoluble property differentiates it from most other currently available biodegradable plastics that have undergone hydrolytic degradation [9]. Indeed, the mechanical properties of this polymer are comparable to those of petroleum-based polypropylene [10]. Nevertheless, mass production of PHB products has been limited to myriad challenges due to its narrow thermal processing window [11]. Thermal degradation could occur when PHB is exposed to a higher temperatures during the manufacturing process [12]. The utilization of this polymer has become potential in biomedical applications due to its biocompatibility and biodegradability [13]. The manufacturing of such biomedical applications can be done via additive manufacturing (AM) technology. At the moment, AM technology (better known as 3D printing) has been applied in functional biomaterials for tissue engineering, fabrication of anatomical and pharmacological models, and production of medical instruments [14,15,16].

AM technology is a process of depositing materials by layering to construct a three-dimensional (3D) object by using a computer-aided design (CAD) file where novel customization can be utilized [17]. It has become one of the key components that have been highlighted in the Industrial Revolution 4.0 (IR4.0), as it is considered the future of the manufacturing sector [18]. The process of additive manufacturing has been categorized into six types: vat-photopolymerization, material extrusion, powder bed fusion, material jetting, directed energy deposition, and sheet lamination [19,20,21]. Each of these categories has its own type of materials, advantages and drawbacks that should be given greater attention so they can be fully utilized according to the proposed applications. The commonly used biomaterials for AM are polymers and composites due to their diversity and high compatibility with different types of AM processes. The polymer materials are usually used in the form of solutions for vat-photopolymerization, thermoplastic filaments for material extrusion and powder beads for powder-bed fusion. Most leading polymers used for 3D printable biomaterials are polylactic acid (PLA), acrylonitrile butadiene styrene (ABS), polyurethane (PU) and polycaprolactone (PCL) [22,23,24]. However, the choices for biocompatible materials in AM technology are still limited. Thus, studies towards broadening those choices of materials could be a great advancement in the medical sector by integrating AM technology. Hence, the selection of AM technique and its materials are crucial to ensure the products complement the specifications needed for the applications.

Currently stereolithography (SLA), which falls under the vat-photopolymerization technique in AM processes, can produce parts with high dimensional accuracy with very intricate details [25]. Thus, it is the most favorable method used in the medical field, such as in surgical tools, temporary replacement medical devices, and fracture-bone casts [26]. It uses a single laser to cure light-sensitive polymer (photopolymer) directed at a particular point, building up layer upon layer contained in a vat/tank. SLA comprises three main components: the printer, material, and CAD file [27]. Any adjustment of these components will give a different outcome to the mechanical properties of 3D structures, especially on the materials side, and particularly the photopolymer resin.

The SLA process is a 3D printing technique based on the principle of resin photo-polymerization. Photopolymer resin comprises monomers, oligomers, and photo-initiators. During the photo-polymerization process, the laser will activate the photo-initiators to release free radicals, which will then induce cross-linking reactions between the functionalized monomers and oligomers to create a solidified structure [28]. Several commonly used monomers are bisphenol A-glycidyl dimethacrylate (Bis-GMA), bisphenol A ethoxylated dimethacrylate (Bis-EMA), urethane dimethacrylate (UDMA) and triethylene glycol dimethacrylate (TEGDMA) [29]. Since the after-products of degradation of Bis-GMA and Bis-EMA generate bisphenol A (BPA), which has been proven to have an estrogenic effect on human health, UDMA has been developed with BPA-free formulation [30]. The polymerization rate in UDMA resin was the highest, even though TEGDMA obtained the highest degree of double bond conversion (DC). The resulting more-robust polymer network exhibited the highest flexural strength compared to TEGDMA. The better mechanical properties presented by UDMA were probably attributed to the cross-linking of stronger hydrogen bonding and less cyclization [31].

UDMA resin could be utilized for medical application purposes by integrating with SLA. SLA demonstrates greater versatility and has the highest fabrication resolution, which is crucial in medical applications [32]. In addition, its customizability to create a custom-fitted design to maintain the fracture bone alignment according to the patients has captivated many researchers to explore the potential of this technology to be embedded in the medical applications [33]. Studies have proven that 3D printed casts could minimize interference, reduce the risk of pressure-related complications and improve ventilation [34]. However, the idea of this research only focused on the material itself.

In treating fractured-bone patients, it is crucial to encase partially, or in a surrounding rigid form called a cast. The immobilized limbs are usually encased by a rigid structure for long periods, often for as long as six weeks or more. However, due to prolonged pressure and poor ventilation of conventional casts, patients have a high tendency toward irritation and muscle fatigue [35]. Moreover, by employing 3D printing technology, the idea to create customized casts for patients that are properly fitted and have a vented structure could be an effective alternative to tackle these problems. To our concern, the integration of biopolymer PHB in the UDMA resin by utilizing 3D printing techniques for casting has not been explored yet. This research aims to study the 3D printability and mechanical properties of PHB/UDMA resin blends and their potential in medical applications as casting for fractured-bone patients.

## 2. Materials and Methods

### 2.1. Materials

Polyhyroxybutyrate (PHB) powder was obtained from Biomer Incorporation (Krailing, Germany), referenced P309, and used as received. Urethane dimethacrylate (UDMA) based resin was purchased from Formlabs Incorporation (Formlabs, Somerville, MA, USA). Isopropanol (IPA) used for cleaning the residual after printing the sample was obtained from Sigma Aldrich (Burlington, MA, USA).

### 2.2. Preparation of PHB/UDMA Resin Blends

Four compositions of weight ratio of polyhydroxybutyrate (PHB): 0, 3, 7, 11 wt.% were incorporated within the urethane dimethacrylate (UDMA) based resin as shown in Table 1. The PHB powder was put inside the vacuum oven for 24 h to remove any moisture. Then, PHB powder and UDMA resin were weighed by using analytical balance according to the weight percentage of compositions that had been decided. They were stirred and stored in the amber veils to prevent any light exposure. The mixture solution was stirred using a magnetic stirrer (WiseStir MSH-20D, Witeg, Germany) at a lower rotation per minute (rpm) at 300 rpm to discard any possibilities for bubble formation. The solutions were left for 24 h under stirring to achieve a homogenous mixture of all components. The resulting mixture was then cooled to room temperature for about ten minutes prior printing.

### 2.3. 3D Printing Design

The design of the 3D printing model was constructed in CAD software (Blender, Amsterdam, The Netherlands). A dog-bone shape (63.5 mm × 9.53 mm × 3.2 mm) was used to evaluate the tensile properties according to ASTM D638 (Type V), whilst a rectangular shape with V-notched (63.5 mm × 12.7 mm × 3.2 mm) was used for impact properties according to ASTM D256. Then, a circle shape (diameter = 10 mm; thickness = 1.5 mm) was used for Fourier transform infrared (FTIR) (Thermo Fisher Scientific, Waltham, MA, USA), field emission scanning electron microscopy (FESEM) (Jeol JSM-IT800 Schottky IT800, Tokyo, Japan) and thermogravimetric analysis (TGA) (TGA/DSC 3+ Mettler Toledo, Columbus, OH, USA); meanwhile, a rectangular shape (19.5 mm × 19.5 mm × 0.5 mm) was used in X-ray diffraction (XRD) analysis (Rigaku Miniflex 600, Tokyo, Japan). The completed design was then rendered and exported into a standard triangulated language (STL) file. Photon S Slicer was used to convert the STL file into a readable file for the SLA 3D printer (Anycubic Photon S, Shenzhen, China) prior to printing.

### 2.4. 3D Printing of PHB/UDMA Resin Blends

**Figure 1:**
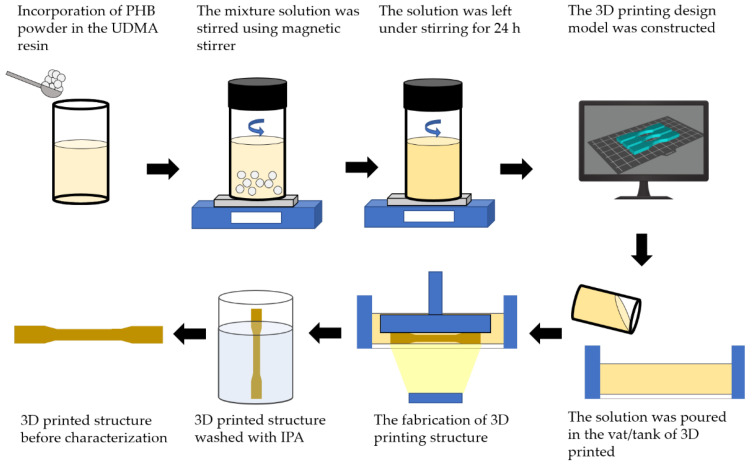
The overview process flow of fabrication of 3D printed PHB/UDMA for characterization.

Triplicate samples were printed for each composition by one-time printing using an SLA 3D Printer with an exposure time of 60 s. The samples were cleaned with isopropanol (IPA) to remove any residual resin that had not fully cured on the surface of the samples. The samples were then cured for 60 m at 60 °C in an ultraviolet (UV) cure machine (Form Cure, Formlabs, Somerville, MA, USA). A total of 24 samples for each tensile and impact test were printed. The samples were then aged in a desiccator for a day (12 samples) and 30 days (12 samples) to evaluate the mechanical properties of 3D printed PHB/UDMA. All other analyses (FTIR, FESEM, XRD, TGA) were performed after a day of aging in desiccator. The overview process flow is shown in Figure 1.

### 2.5. Viscosity Measurement for Pure UDMA and PHB/UDMA Resin Blends

The viscosity of the pure UDMA resin and PHB/UDMA resin blends were measured by using a rheometer (DVNext Rheometer, AMETEK Brookfield, Middleborough, MA, USA). The spindle used was RV-04 set at 50 rotations per minute (rpm). All the measurements were taken at room temperature.

### 2.6. Tensile Test

Tensile tests were performed using the dog-bone samples based on Type V of ASTM D638 (63.5 mm × 9.53 mm × 3.2 mm) using a universal testing machine (Instron 5566, Instron Corporation, Norwood, MA, USA) with a crosshead speed of 5 mm/min and equipped with 10 kN load cell. The tests were conducted at ambient temperature and 50% of relative humidity until the failure of the samples and the stress-strain curve was obtained.

### 2.7. Impact Test

Impact tests were measured using a rectangular shape with a V-notch according to ASTM D256 (63.5 mm × 12.7 mm × 3.2 mm) by using an Impact Test with Notcher (Instron Ceast 9050, Instron Corporation, Norwood, MA, USA) with nominal impact energy at 11 J and impact velocities of 3.5 m/s.

### 2.8. Fourier Transform Infrared (FTIR)

Fourier Transform Infrared (FTIR) spectrum test was performed to obtain specific information about chemical bonds and molecular structure of PHB powder, UDMA resin, 3D printed UDMA, and 3D printed PHB/UDMA blends composites. An FTIR spectrometer (Nicolet iS50, Thermo Fisher Scientific, Waltham, MA, USA) was used to analyze the changes in spectra within a range of 400 cm^−1^–4000 cm^−1^. The degree of double bond conversion (DC %) was obtained for each compositions by using Equation (1) [36]:

(1) $$\mathrm{DC}\;(\%)=\left[1-\left(\frac{{(1638\;\mathrm{cm}}^{-1}{/1608\;\mathrm{cm}}^{-1})\mathrm{polymer}}{{(1638\;\mathrm{cm}}^{-1}{/1608\;\mathrm{cm}}^{-1})\mathrm{monomer}}\right)\right]\;\times \;100$$

### 2.9. Field-Emission Scanning Electron Microscopy (FESEM)

The morphology of 3D printed UDMA and PHB/UDMA blends surface were studied using Field Emission Scanning Electron Microscopy (Jeol JSM-IT800 Schottky IT800, Tokyo, Japan). The observation and microphotographs for all compositions of 3D printed samples were taken at 1K resolution.

### 2.10. X-ray Diffraction (XRD)

The structural characterization of the PHB powder, 3D printed UDMA and PHB/UDMA were performed by an X-ray diffraction instrument (Rigaku Miniflex 600, Tokyo, Japan). The X-Ray diffractometer was regulated at 40 kV and 15 mA, with Cu_Kα_ radiation (λ = 0.154 nm). The scattering angle was set from 2.5° to 30° whilst the step duration was set at 10° s^−1^. All the peaks were observed and identified in concordance with the International Center for Diffraction Data (ICDD). The crystallinity index for each composition of 3D printed samples was obtained using OriginPro (OriginLab Corporation, Northampton, MA, USA) according to the Equation (2):

(2) $$\mathrm{Crystallinity}\;\mathrm{Index}\;\left(\%\right)=\frac{\mathrm{Area}\;\mathrm{of}\;\mathrm{all}\;\mathrm{the}\;\mathrm{crystalline}\;\mathrm{peaks}}{\mathrm{Area}\;\mathrm{of}\;\mathrm{all}\;\mathrm{the}\;\mathrm{crystalline}\;\mathrm{and}\;\mathrm{amorphous}\;\mathrm{peaks}}\;\times \;100$$

### 2.11. Thermogravimetric Analysis (TGA)

Thermal stability of PHB powder, 3D printed UDMA and 3D printed PHB/UDMA were studied using a thermogravimetric analyzer by observing the weight loss of the samples upon heating (TGA/DSC 3+ Mettler Toledo, Columbus, OH, USA). The measurements were conducted in a nitrogen, N_2_ atmosphere at a constant flow rate of 20 mL/min, the heating scan rate of 20 °C/min, and the temperature was elevated from 25 °C until 700 °C.

### 2.12. Statistical Analysis

Statistical analysis of tensile properties (Young’s modulus, tensile stress and tensile strain) and impact strength values were performed through the Statistical Package for the Social Sciences (SPSS v.21) (IBM Corporation, Armonk, NY, USA). As the data were found to be consistent with a normal distribution (*p* > 0.05), the data were then analyzed using two-way analysis of variance (ANOVA) to study the interaction between the varied composition of PHB (wt.%) incorporation within UDMA resin and the aging duration of 3D printed PHB/UDMA towards mechanical properties.

## 3. Results and Discussion

### 3.1. The Effect Parameters on 3D Printing

**Figure A1:**
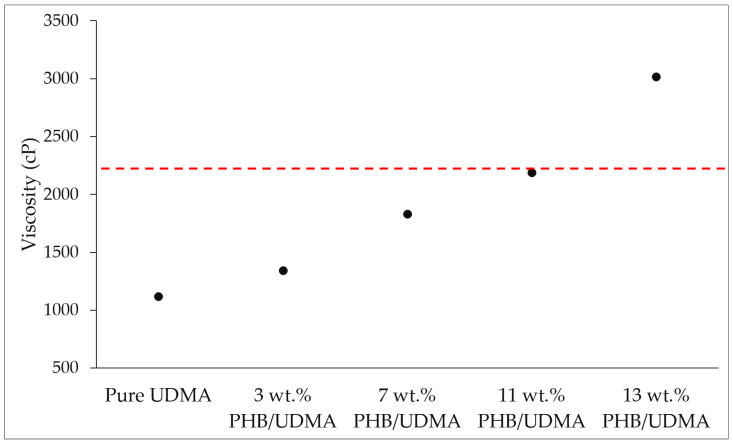
Viscosities of pure UDMA resin and PHB/UDMA resin blends at room temperature.

The viscosity of the resin plays a crucial role to the 3D printability of the structure. However, commercialized resins have their specific viscosity. Since PHB powder has been incorporated into the UDMA resin, its viscosity (PHB/UDMA resin blend) had increased with the increase in PHB content (Appendix A, Figure A1). Higher concentrations of PHB tend to form aggregates in the resin, significantly increasing their viscosity [37]. Pure UDMA resin has a viscosity at 1120 cP at room temperature. The PHB/UDMA resin blends contained with 11 wt.% of PHB reached a value of 2188 cP. It was the maximum viscosity of the PHB/UDMA resin blend that has been successfully printed. The 13 wt.% PHB/UDMA resin blends were unable to be printed due to their high viscosity, which was 3016 cP.

**Figure 2:**
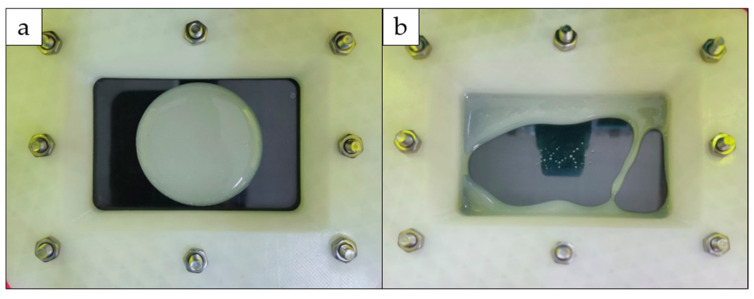
13 wt.% of PHB/UDMA resin blends: (**a**) Before printing; (**b**) After printing.

It is observed that the limitation of printability was approximately 2300 cP. Upon exceeding the limitation, the PHB/UDMA blend solution become very viscous, which then restricted the flow of the mixtur solution into the center of the vat/tank. Due to this factor, the amount of resin flowing is insufficient for the material to be printed. Figure 2 shows the condition of 13 wt.% PHB/UDMA resin blends in the vat of 3D printer before and after printing.

The exposure time of UDMA resin towards ultraviolet light also influenced the 3D printed structure. Commercialized resins have their own specific exposure rate time setting. As PHB powder was added to the resin, it also disrupted the actual exposure time setting. After optimization, the optimal parameters for the setting were obtained (Appendix A, Table A1).

### 3.2. Tensile Properties

According to the Shapiro–Wilk test, the data of tensile properties are normally distributed since all the compositions of 3D printed PHB/UDMA showed a significant value of *p* > 0.05. Two-way ANOVA statistical analyses were executed to study the significance of the composition of PHB (wt.%) incorporated within UDMA resin, and the aging duration of varied 3D printed PHB/UDMA towards tensile properties. The effect of the composition of PHB (wt.%) incorporation within UDMA resin, effect of aging duration and effect of the interaction between both of them, were denoted as A, B and A × B, respectively. The interaction effects of those variables on Young’s modulus, tensile stress and tensile strain were examined independently. The results of two-way ANOVA are given in Table 2. F and *p* values are crucial to establish null hypotheses. If the F-value shown in Table 2 is greater than the F-value determined by the critical F-value by using F-table, null hypotheses are rejected [38,39]. The validity of the null hypotheses is determined by the *p*-value. For example, in this study when the effect of the composition of PHB (wt.%) incorporation within UDMA resin on Young’s modulus was investigated, degrees of freedom (DF) in the numerator was obtained as 3, whilst the DF for the denominator was 8. By using F table, critical value was determined as 4.27. The F-value observed in Table 2 (=7.34) is larger than the critical F value (=4.07) at α = 0.05. In this case, null hypotheses can be rejected.

After implementing the same procedure on duration aging of varied 3D printed PHB/UDMA as well as the interaction between both of them, F and *p*-value of these two independent variables towards tensile properties (Young’s modulus, tensile stress and tensile strain) were determined. Data given in Table 2 indicate that the composition of PHB (wt.%) incorporation within UDMA resin, the aging duration and the interaction between both of them have significant effects on Young’s modulus, tensile stress and tensile strain.

**Figure 3:**
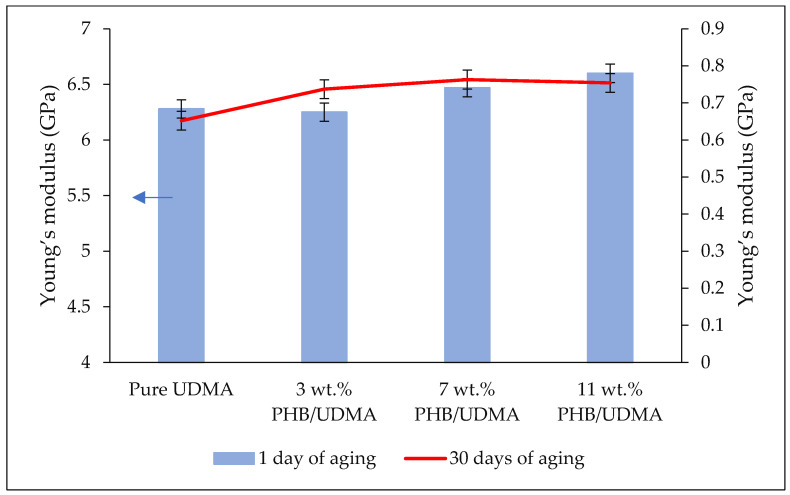
Comparative results of Young’s modulus of varied 3D printed PHB/UDMA after a day and a month of aging.

Figure 3 shows the comparison result of Young’s modulus for varied 3D printed PHB/UDMA. Young’s modulus is defined as the ratio between stress and strain at the elastic stage of the tensile measurement or better known as the stiffness of a material. As expected, a slight improvement of Young’s modulus could be observed at higher wt.% of PHB powder (particles) incorporation within UDMA resin (polymer matrix). The 11 wt.% PHB/UDMA had achieved 6.6 GPa of Young’s modulus, an increment of 5% compared to the 3D printed pure UDMA. Many studies proved that the addition of micro/nanoparticles have significant improvement. In most particulate-polymer composites, hard particles have much stiffer values rather than the matrix. As PHB powder is a semi-crystalline polymer, its Young’s modulus crystallinity mostly contributed towards the enhancement of the stiffness of the 3D printed PHB/UDMA.

**Figure 4:**
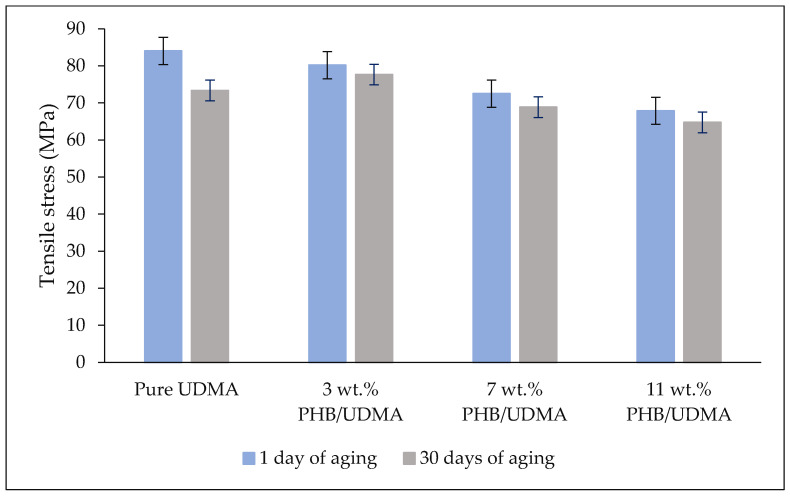
Comparative results of tensile stress of varied 3D printed PHB/UDMA after a day and a month of aging.

Figure 4 shows the comparative results of tensile stress of varied 3D printed PHB/UDMA, where 3D printed PHB/UDMA exhibited lower tensile stress than 3D printed pure UDMA. The tensile stress of particulate-polymer composites are highly influenced by three factors, which are: particle size of filler, particle/matrix interfacial adhesion and particle loading [40]. In these findings, PHB powder acts as filler particles. As PHB powder increased, it would have detached from the UDMA matrix due to the weak interfacial adhesion between them. Hence, stress transfer between PHB particles and UDMA matrix became inefficient due to the poor bonding between them. Therefore, tensile stress reduction occurred by adding the PHB powder. Moreover, the filler particles tend to agglomerate resulting in random stress distribution. Consequently, the main crack was initiated due to the inevitably large size of voids that formed, thus decreasing the tensile stress [41]. It is reported that, as the agglomeration of filler-particle increased, the distribution of applied stress became irregular and thereby initiated local stress concentrations. Hence, the cracks will be formed around the stressed region of the filler–matrix interface [42].

Even though the incorporation of PHB powder weakened the UDMA, it seems that the PHB could retain the tensile strength after a month of aging in the desiccator at room temperature. After a month of aging, the 3D printed UDMA had decreased to almost 10% of its tensile strength. However, the incorporation of PHB within the UDMA matrix seemed to successfully retain its tensile strength as the 3D printed of 11 wt.% PHB/UDMA only showed a decrease of below 2% from its tensile strength.

**Figure 5:**
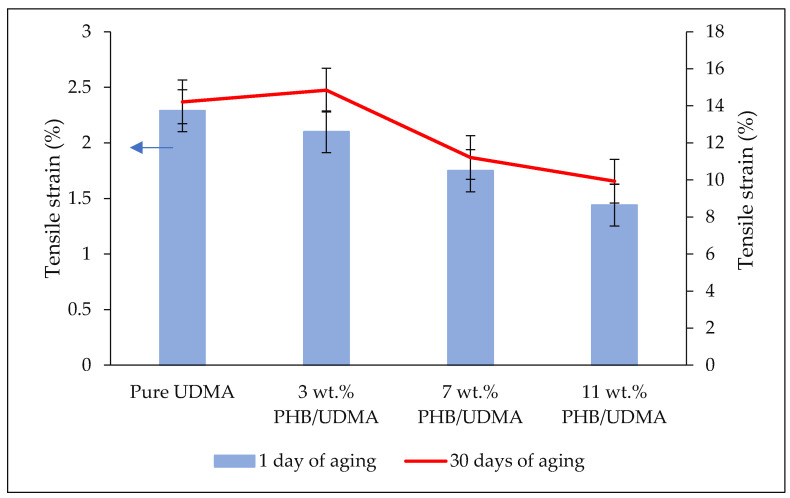
Comparative results of tensile strain of varied 3D printed PHB/UDMA after a day and a month of aging.

Figure 5 shows that the comparative tensile strain values of varied 3D printed PHB/UDMA. The strain decreases steadily with the increase in PHB powder incorporation. The lowest tensile strain recorded at 11 wt.% PHB/UDMA at 1.44%, meanwhile for the 3D printed pure UDMA it recorded the highest strain at a value of 2.30%. The reduction in strain was mainly attributed to the lack of deformability of rigid interphase between PHB powder and the UDMA resin. Many studies have reported that the lower strain in filled polymer composites was due to the deformation of the filler being much lower than the polymer matrix. Hence, the filler restricted the polymer matrix to deform more than the overall deformation of the composites [43].

### 3.3. Impact Strength

The Shapiro–Wilk test did not show evidence of non-normality towards the impact strength for all the varied 3D printed PHB/UDMA. Thus, two-way ANOVA was conducted to observe the significant effect of the composition of PHB (wt.%) incorporated within UDMA resin and the aging duration of varied 3D printed PHB/UDMA towards impact strength. Two-way ANOVA results of impact strength of the varied 3D printed PHB/UDMA are shown in Table 3. The composition of PHB (wt.%) has significant effect towards impact strength where *p* < 0.05 (=0.001), however there were not statistical differences for the aging duration of varied 3D printed PHB/UDMA where *p* > 0.05 (=0.191). Thus, there were also not statistically significant differences for the interaction between those two independent variables towards impact strength.

**Figure 6:**
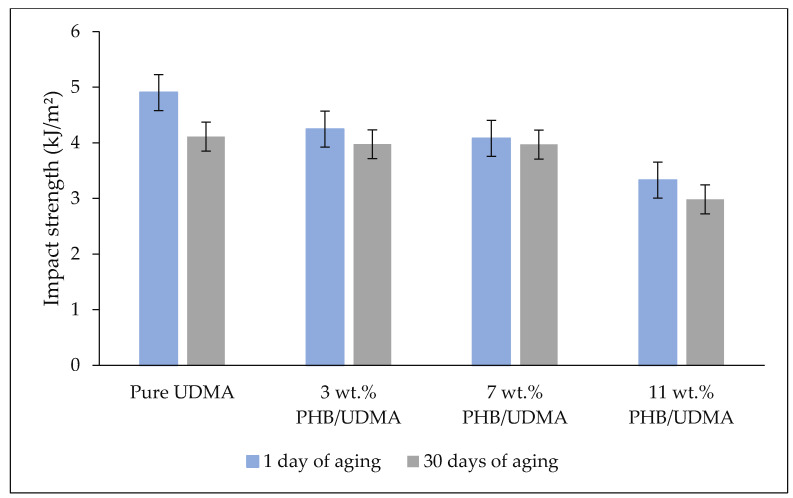
Impact strength of varied 3D printed PHB/UDMA.

Figure 6 shows the comparative impact strength values of varied 3D printed PHB/UDMA. The impact strength of 3D printed PHB/UDMA was lower compared to the 3D printed pure UDMA. The agglomeration of filler induced random stress distribution, initiating cracks at a certain region within the composites. Moreover, as the PHB content was increased, the molecular chain of the amorphous phase within the UDMA matrix was restricted. Hence, the decrease in the impact strength was expected as the PHB loading increased.

The agglomeration of PHB particles which were acting as filler within the UDMA matrix caused the reduction in impact strength as the PHB contents increased. In composites, the agglomeration is a region that could act as a foreign body. When there were increments of agglomerates at higher PHB loading, PHB became obstacles that restricted the mobility of the UDMA molecular chain which later induced failure under stress. An increase in filler loading does not eventually strengthen the composites. In a certain case, it could weaken the materials, since more defects were created as the filler fractions become higher. Moreover, it is important to elucidate that brittleness of the composites was attributed to the resistance of PHB agglomerations against UDMA deformation. As a result, the propagation of cracks became much faster due to the lack of plastic deformation and failure to absorb energy [44]. Thus, the impact strength of the particulate composite is highly affected by the filler volume fraction.

In addition, the impact strength of the composite is also mainly attributed to its particle size, adhesion to the matrix, and the uniformity of their distribution in the polymer composite [45]. The addition of natural fillers to the polymer matrix increased the brittleness as portrayed by the impact test.

### 3.4. Fourier Transform Infrared Spectroscopy (FTIR)

**Figure 7:**
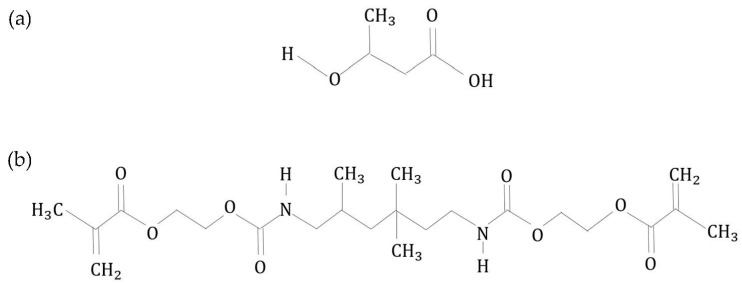
The molecular structure: (**a**) PHB and (**b**) UDMA.

**Figure 8:**
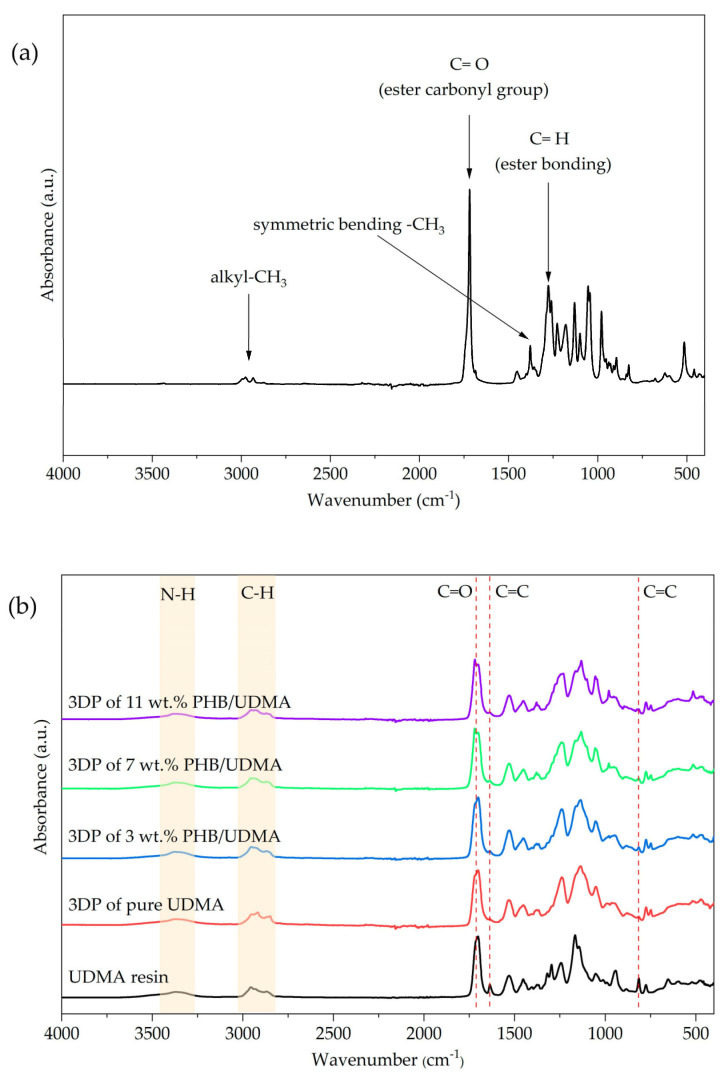
Infrared spectra of: (**a**) PHB powder; (**b**) UDMA resin; 3D printed UDMA and 3D printed PHB/UDMA.

For PHB powder, the peaks observed agreed with those found in previous studies. The ester carbonyl group peak is located at 1729 cm^−1^ and corresponds to the C=O stretching modes in the molecular chain. Adsorption band at the C–H group (ester bonding) can be found at 1279 cm^−1^. Meanwhile, C–O stretching (ester bonding) depicted a series of bands from 1163 cm^−1^ until 1210 cm^−1^. The bending modes of methyl group appeared at 2969 and 2927 cm^−1^ whilst a peak at 1377 cm^−1^ depicted its symmetric bending. The asymmetric bending of –CH_3_ and –CH_2_ is located at 1452 cm^−1^. Lastly, a weak band of absorbance peak depicted at 3434 cm^−1^ corresponds to the hydroxyl group. The molecular structure of PHB and infrared spectrum of PHB powder are shown in Figure 7a and Figure 8a, respectively.

The chemical nomenclature of UDMA is 1,6-bis-(metalocriloxi-2-etoxicarbolamino)-2,4,4-trimethylexane. It contains an aliphatic core and two urethane links. The sharp absorbance peak at 1709 cm^−1^ corresponds to the C=O stretching whilst the C=C stretching mode is depicted at 1637 cm^−1^. Meanwhile, a wider peak showed between 3200 cm^−1^ and 3400 cm^−1^ concordant with the N-H stretching mode, whilst the bending of N-H was located at 1634 cm^−1^. Lastly, the C-H stretching mode is located in the range of 2800 cm^−1^ and 3000 cm^−1^. The molecular structure of UDMA is shown in Figure 7b, meanwhile Figure 8b shows the infrared spectra for UDMA resin and varied 3D printed PHB/UDMA.

The structure-property of the dimethacrylate polymer network can be elucidated further by determining the degree of double bond conversion (DC). The peak located at 1637 cm^−1^ and 816 cm^−1^ corresponds to the stretching modes of the vinyl group and the twisting of the carbon–carbon double bond, respectively. The intensity of these peaks showed the amount of the vinyl double bonds that remained after the samples were cured with the light. Even though both peaks have been utilized for the determination of the acrylates and methacrylates polymerization, the absorption peak depicted at 1637 cm^−1^ has been selected as the bond is stronger than the latter one, and due to that, it is the most commonly used.

In poly(dimethacrylate), the DC could not achieve at full capacity [46]. The characterization of polymer network by DC that resulted below 50% is invalid in practical applications due to the sol fraction formation [29]. The degree of DC in 3D printed UDMA is much higher than that of 3D printed PHB/UDMA shown in Table 4. However, only 3 wt.% PHB/UDMA obtained 51.17% of DC. For 7 and 11 wt.% of PHB loading, the minimum degree of double bond conversion has surpassed the minimum value for the clinical application. A previous study reported that the recommendation for the clinical application should be at least 55% of DC in the polymer network [47]. The detachment of the uncured resin, or better known as leaching, could trigger inflammation in the tissues and significantly affect towards organism [48].

### 3.5. X-ray Diffraction (XRD)

**Figure 9:**
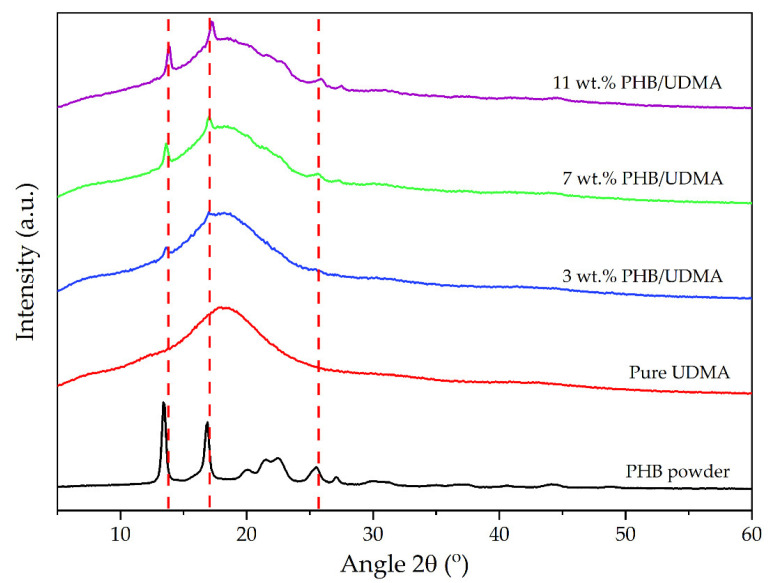
X-ray diffraction patterns of PHB powder, 3D printed UDMA and 3D printed PHB/UDMA.

Figure 9 represents the XRD patterns for PHB powder, 3D printed pure UDMA and 3D printed PHB/UDMA. As was expected, no sharp peak belongs to the 3D printed UDMA as it is an amorphous solid material. The XRD pattern of PHB powder depicted a series of crystalline peaks that had been recognized by the Joint Committee on Powder Diffraction Standards (JCPDS) located at 13.5°, 16.9°, and 25.5°. The unit cell of PHB has an orthorhombic system crystalline structure. The peaks at the 2θ = 13.5°, 16.9°, and 25.5° were also detected in the XRD patterns of 3D printed PHB/UDMA, which were recognized to be (020), (110), and (130), respectively [49]. This proved that the incorporation of PHB within the amorphous UDMA resin induced the crystallinity of the 3D printed PHB/UDMA.

As the content of filler increases, more crystalline peaks appear in the 3D printed PHB/UDMA. The hypothesis was proven as there were clear sharp peaks present when the PHB content increased. The crystallinity index (CI) for the PHB powder was 91.52%, whilst for the 3 wt.% PHB/UDMA showed about 10.34% of CI. Eventually, 11 wt.% PHB/UDMA portrayed the highest CI for the 3D printed composites at 34.98%. This result proved that the brittleness of the 3D printed PHB/UDMA was increased as the sharp peaks that belong to the PHB became obvious as the PHB contents increased. Table 5 shows the CI for the varied 3D printed PHB/UDMA.

### 3.6. Field Emission Scanning Electron Microscopy (FESEM)

**Figure 10:**
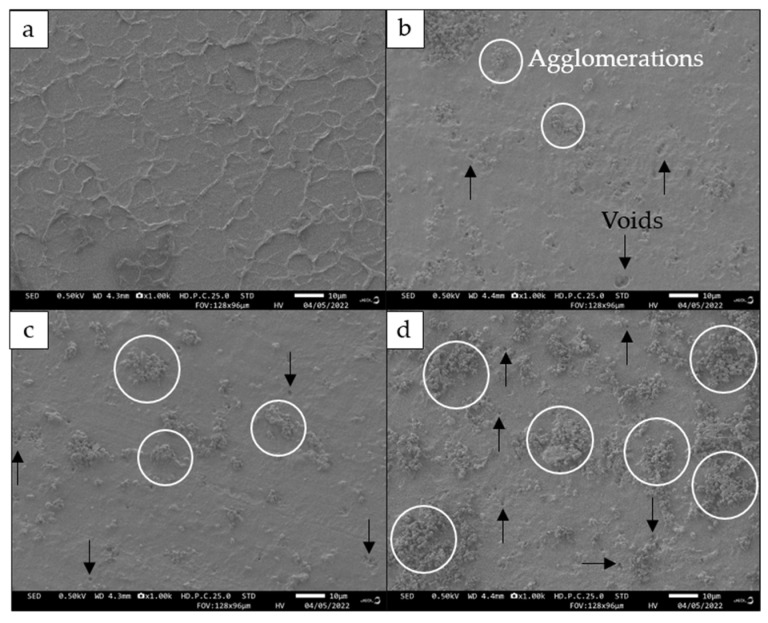
FESEM microphotographs of 3D printed: (**a**) UDMA; (**b**) 3 wt.% PHB/UDMA; (**c**) 7 wt.% PHB/UDMA and (**d**) 11 wt.% PHB/UDMA.

FESEM microphotographs of 3D printed pure UDMA and 3D printed PHB/UDMA are shown in Figure 10. The surface of 3D printed pure UDMA is smooth with a laminar-like structure, whereas for the 3D printed PHB/UDMA it portrayed irregular microspheres of approximately 1 μm in size. PHB powder was deposited within the UDMA resin and shrouded in a bigger region as its content increased. As the PHB loading was increased, more agglomerations could be observed, and thus it can be correlated with the decrease of the tensile strength 3D printed by PHB/UDMA. As the PHB contents increased, more voids or micro-cracks can be seen. This might be contributed to the scarcity of uniform dispersion between PHB powders and the UDMA resin.

In most particulate composites, the particles of filler are not uniformly distributed. The composites have local domains where the particles are clustered together [50]. When the PHB particles agglomerate in clusters, the diameter of its phase increases. Thus, the effects of aggregations of particles as clusters can be correlated with the decrease in tensile and impact strength of 3D printed PHB/UDMA. Experimental findings show that there is a strong correlation between the strength of 3D printed PHB/UDMA and the distribution fraction of PHB particles. Hence, the results were in good agreement with the clustering effect of PHB particles that act as fillers towards the mechanical properties.

### 3.7. Thermogravimetric Analysis (TGA)

**Figure 11:**
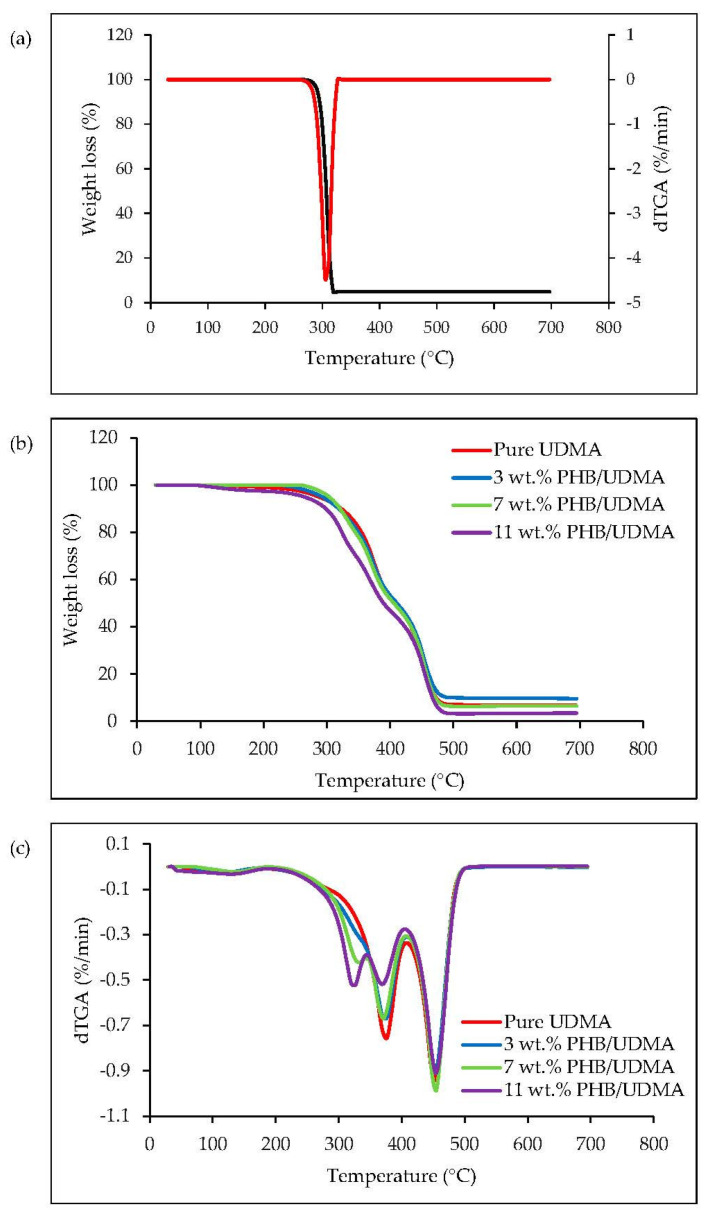
The thermal graphs of: (**a**) TGA and dTGA of PHB powder; (**b**) TGA curves of 3D printed PHB/UDMA and (**c**) dTGA curves of 3D printed PHB/UDMA.

It can be observed that 3D printed UDMA exhibited a two-step degradation mechanism. It was expected for the photo-polymerization resin composites to have two or three mass loss steps during decomposition [51]. For the PHB powder, it was observed that only a thermal event occurred started at 262 °C and up to 326 °C with a weight loss at 95.06%. The maximum peak of degradation rate for PHB powder depicted at 305 °C as shown in Figure 11a.

Figure 11b shows the thermogravimetric analysis (TGA) curves of varied 3D printed PHB/UDMA, where 3D printed UDMA portrayed two steps of thermal degradation started at 262 °C and completed at 496 °C, leaving some solid residue at 6.65%. The first maximum in the degradation rate occurred at 376 °C, whilst the second was at 454 °C. The first degradation involved with the end-chain scission (vinylidene end groups) was attributed to the termination by disproportionation reaction during polymerization. The latter degradation was induced by the random scission at abnormal head-to-head linkages.

As the PHB contents were increased, an additional peak gradually started to appear for 3D printed PHB/UDMA as shown in Figure 11c. Therefore, for the 3D printed PHB/UDMA it consisted of three steps of thermal degradation. The first peak of maximum degradation rate for 3D printed PHB/UDMA started to shift to the lower temperature as the PHB contents increased. They showed at 341 °C, 335 °C, 327 °C for 3, 7 and 11 wt.% PHB/UDMA, respectively, as shown in Table 6.

### 3.8. Application for 3D Printed Casting

The process began with the 3D scanning of the broken arm to obtain the ergonomic structure. The model patient required to stay still at least 60 s for the scanning to take place. The speed for the acquisition of the anatomy patient’s hand was nonetheless a fundamental procedure to maximize the precision and accuracy of the 3D images that would be obtained. The steadiness of the actual patient would become the biggest challenge; however, it was noted that this research only focused on the feasibility of its printability, mechanical properties and its potential for the fractured-bone patient.

The 3D images obtained from the scanning were transposed in the 3D modelling software (Blender, Amsterdam, The Netherlands). The 3D model file was further refined at a certain region that could not be fully scanned since the movement of the patients’ hand disrupted the actual design. After 3D reconstruction and editing of the 3D model file had finished, the file was then converted into an STL file format that was compatible with the SLA 3D printer. The optimal support was defined at the vulnerable areas and to the exact limb size for a snug fit prior to printing.

The 7 wt.% of PHB/UDMA resin blends formulation was selected to print the cast as it the most recommended based on its mechanical properties, structure, crystallinity and thermal evaluation concordance with the preceding analysis that had been made. The printing exposure time was set at 60 s, as that was the optimized setting according to the previous evaluation to ensure a fine cast could be printed (Appendix A, Table A2). The weight of the PHB/UDMA resin blends required to 3D print a cast was approximately 50 g. The cast was further cleaned with the IPA to remove any residue of uncured resin. Then, it had been cured using a cure machine for about an hour at 60 °C.

**Figure 12:**
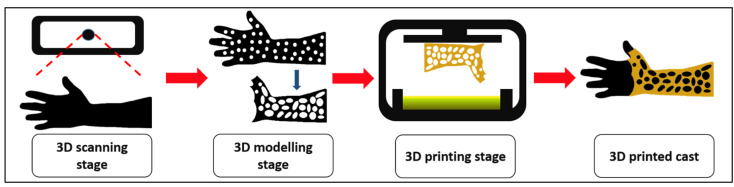
The development of 3D printed cast.

**Figure 13:**
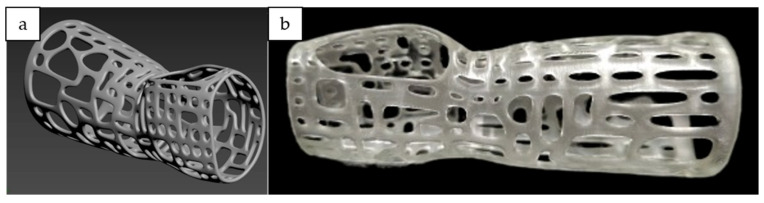
(**a**) The 3D model of cast; (**b**) 3D printed cast.

The traditional casting for fractured-bone patients has caused discomfort to them as the cast used had poor ventilation and did not fit properly according to the needs of patients. The 3D printing technology has been integrated to produce a cast with the aim of creating a cast that comes with the features that can mitigate these issues. Therefore, a ventilated and lighter 3D printed cast had been successfully manufactured. The flow processes of the 3D printed cast are shown in Figure 12. The CAD model of the cast is shown in Figure 13a whilst the 3D printed cast is shown in Figure 13b.

## 4. Conclusions

This study focused on the 3D printability and mechanical properties of materials that could be utilized in medical applications. The chemical reaction that occurs at the double bonds in acrylates or methacrylate induced by the free radical mechanism performed by the photo-initiator, will be manifested in terms of 3D structure when exposed to light. The printable UDMA resin with a composition of up to 11 wt.% of PHB powder loading was developed for SLA 3D printing. Since PHB did not contain any of those functional groups, the UDMA-based resin could complement each other since PHB could retain its mechanical properties over the month. The tensile strength 3D printed at 11 wt.% PHB/UDMA only decreased by about 2% of its strength after a month of aging. The pure 3D printed UDMA decreased significantly up to almost 10% of its strength after a month of aging. A 3D printed cast based on 7 wt.% PHB/UDMA resin blend formulation was successfully developed. The material could be utilized for temporary medical devices such as casting for the fracture-bone patient.

## Figures and Tables

**Table 1:** Sample compositions of PHB/UDMA resin blends.

Samples Name	Composition of Samples		
	PHB (wt.%)	PHB (g)	UDMA (g)
Pure UDMA	0	0.00	15.00
3 wt.% PHB/UDMA	3	0.47	15.00
7 wt.% PHB/UDMA	7	1.13	15.00
11 wt.% PHB/UDMA	11	1.86	15.00

**Table 2:** Two-way ANOVA results of varied 3D printed PHB/UDMA towards tensile properties.

Two-Way ANOVA	Young’s Modulus			Tensile Stress			Tensile Strain		
	A	B	A × B	A	B	A × B	A	B	A × B
DF	3	1	3	3	1	3	3	1	3
SS	0.183	193.04	0.089	699.88	129.18	65.74	32.74	678.83	17.50
MS	0.061	193.04	0.030	233.29	129.18	21.91	10.91	678.83	5.84
F-value	7.34	23,277.33	3.57	44.16	24.45	4.15	15.02	934.19	8.03
*p*-value	0.003	0.000	0.038	0.000	0.000	0.024	0.000	0.000	0.002

**Table 3:** Two-way ANOVA results of impact strength of the varied 3D printed PHB/UDMA.

Two-Way ANOVA	Impact Strength		
	A	B	A × B
DF	3	1	3
SS	6.400	0.435	0.441
MS	2.133	0.435	0.147
F-value	9.134	1.861	0.630
*p*-value	0.001	0.191	0.606

**Table 4:** Peak heights and resulting DC (%) for 3D printed UDMA and PHB/UDMA.

Composition	Absorbance Peak		Degree of Conversion (%)
	1637	1608	
Pure UDMA	0.0404	0.0265	63.73
3 wt.% PHB/UDMA	0.0421	0.0207	51.17
7 wt.% PHB/UDMA	0.0505	0.0274	55.68
11 wt.% PHB/UDMA	0.0382	0.0213	56.86

**Table 5:** Crystallinity index (CI) for PHB powder and 3D printed PHB/UDMA.

Composition	Crystallinity Index (%)
PHB powder	91.52
3 wt.% PHB/UDMA	10.34
7 wt.% PHB/UDMA	26.74
11 wt.% PHB/UDMA	34.98

**Table 6:** Thermal events of thermal degradation and their corresponding of weight loss (%).

Sample	PHB Powder	Pure UDMA	3 wt.% PHB/UDMA	7 wt.% PHB/UDMA	11 wt.% PHB/UDMA
T_1_ (°C)	262–326	262–411	298–348	263–342	262–341
T_2_ (°C)	-	411–496	348–405	342–406	341–405
T_3_ (°C)	-	-	405–510	406–504	405–510
Residual weight	4.95	6.65	9.53	6.47	3.45

## Data Availability

Not applicable.

## Appendix A

**Table A1:** The optimized parameter settings for PHB/UDMA resin blends.

Parameters	Value
Layer thickness (mm)	0.05
Normal exposure time (s)	60.00
Off time (s)	1.00
Bottom exposure time (s)	60.00
Bottom layers	8

**Table A2:** 3D printer setting parameters for 3D printed cast.

Parameters	Values
Layer thickness (mm)	0.05
Normal exposure time (s)	60.00
Off time (s)	1.00
Bottom exposure time (s)	60.00
Bottom layers	8
XY resolution (µm)	47
Y axis resolution (µm)	1.25
Layer resolution (µm)	25–100
